# Air Pollution and Adverse Pregnancy Outcomes: Response

**Published:** 2004-10

**Authors:** Shiliang Liu, Daniel Krewski, Yuanli Shi, Yue Chen, Richard T. Burnett

**Affiliations:** Faculty of Medicine, University of Ottawa, Ottawa, Ontario, Canada, E-mail: sliu@uottawa.ca; Healthy Environments and Consumer, Safety Branch, Health Canada, Ottawa, Ontario, Canada

We thank Bukowski for his critical comments on our article (Liu et al. 2003), in which we reported associations between ambient air pollution and adverse pregnancy outcomes in Vancouver, Canada. In recent years, air pollution has come to be recognized as an important risk factor for a number of adverse health outcomes, particularly cardiorespiratory morbidity (Burnett et al. 1997, 2001; Lin et al. 2002, 2003; Yang et al. 2003) and mortality (Burnett et al. 1997; Dockery et al. 1993; Pope et al. 1995; Villeneuve et al. 2003).

The adverse effects of air pollution on pregnancy outcomes, such as low birth weight (LBW), preterm birth, intrauterine growth retardation (IUGR), and developmental anomalies are of increasing concern. Before our study, there were reports of associations between particulate (total suspended particulate) and gaseous (carbon monoxide, sulfur dioxide, and nitrogen dioxide) air pollutants and adverse pregnancy outcomes from southern California (Ritz et al. 2000, , 2002), China (Wang et al. 1997; Xu et al. 1995), and the Czech Republic (Bobak 2000; Dejmek et al. 1999). Replication of these findings in different populations under different conditions of exposure is an important aspect of epidemiologic research, with consistency of results strengthening the weight of evidence for a true association between exposure and outcome.

Data on important predictors of adverse pregnancy outcomes were not available to us for use in our study (Liu et al. 2003). Although numerous risk factors have been identified (including maternal age, parity, infant sex, and season of birth, as well as gestational age and birth weight, in the case of LBW and preterm birth, respectively, which we were able to take into account), our understanding of the etiology of adverse pregnancy outcomes remains far from sufficient (Kramer 2003). The omission of known or unknown risk factors for birth anomalies may lead to uncontrolled or residual confounding of the association between air pollution and adverse pregnancy outcomes, as Bukowski suggests. However, the extent to which residual confounding might occur in our data is unclear. Schwartz and Morris (1995) have argued that the estimated effects of air pollution are unlikely to be confounded by these factors because they are unlikely to be correlated with daily air pollution levels.

Exposure assessment is always a critical factor in environmental epidemiology (Rothman 1993). Like most other studies of air pollution and population health, our study (Liu et al. 2003) relied on ecologic rather than personal indicators of exposure, with average ambient air pollution concentrations determined using one or more fixed site monitors within census areas in Vancouver. Janssen et al. (1998, 1999) have suggested that air pollution levels from outdoor monitoring stations can provide useful surrogates for personal exposure. Exposure misclassification due to the use of fixed site ambient monitors rather than personal dosimeters is likely to underestimate rather than overestimate the effect of air pollution on birth outcomes (Mallick et al. 2002; Zeger et al. 2000).

The weight of evidence that air pollution is causally related to adverse pregnancy outcomes would be considerably increased through understanding of biological mechanisms by which such effects could occur.

Burkowski notes that we (Liu et al. 2003) included a number of statistical tests of the strength of association between air pollution and adverse pregnancy outcomes, and observes that multiple testing raises the risk of false positives. Our *a priori* strategy for hypothesis testing focused on predetermined stages of pregnancy (month or trimester), which are thought to represent periods of differential susceptibility to exogenous exposures. Findings from both epidemiologic and toxicologic studies suggest that the fetus is most susceptible to the effects of air pollution during the first trimester (Generoso et al. 1987; Rutledge 2000). Human studies also have suggested that initial changes leading to IUGR might be triggered in early pregnancy, around the time of implantation (Duvekot et al. 1995; Khong et al. 1986). Air pollutants may be absorbed into the maternal bloodstream, cross the placental barrier, and have direct toxic effects on the fetus.

Our *a priori* strategy for the development of appropriate risk models focused on single-pollutant models, with adjustment for relevant covariates available to us, as we reported in Tables 4–7 (Liu et al. 2003). Our strategy also called for an assessment of the robustness of the associations between pregnancy outcomes and specific pollutants against adjustment for copollutants. Although this strategy does involve a moderately large number of statistical tests of the significance of logistic regression coefficients associated with specific pollutants, our evaluation of the data is based more on the evidence provided by this set of hypothesis tests as a whole, rather than on the results of individual tests alone.

Overall, our data suggest that adverse pregnancy outcomes are associated with exposures to air pollutants during pregnancy, particularly in early gestation. Because of limitations of our study, we (Liu et al. 2003) concluded that

“these effects require further examination in other populations, and further research also needs to be conducted with more detailed information on personal exposures, effect modifiers, and other adverse pregnancy outcomes such as birth defects and spontaneous abortion.”

Our data need to be interpreted in the context of the emerging body of scientific evidence on air pollution and adverse pregnancy outcomes, to which we have made a contribution.
